# Chemotherapy-Mediated Complications of Wound Healing: An Understudied Side Effect

**DOI:** 10.1089/wound.2023.0097

**Published:** 2024-03-06

**Authors:** Paulina Słonimska, Paweł Sachadyn, Jacek Zieliński, Marcin Skrzypski, Michał Pikuła

**Affiliations:** ^1^ Laboratory for Regenerative Biotechnology, Gdańsk University of Technology, Gdańsk, Poland.; ^2^ Department of Oncologic Surger, Medical University of Gdańsk, Gdańsk, Poland.; ^3^ Department of Oncology and Radiotherapy, Medical University of Gdańsk, Gdańsk, Poland.; ^4^ Laboratory of Tissue Engineering and Regenerative Medicine, Division of Embryology, Department of Anatomy, Faculty of Medicine, Medical University of Gdańsk, Gdańsk, Poland.

**Keywords:** wound healing, chemotherapy, cancer, epigenetic

## Abstract

**Significance:**

Chemotherapy is a primary method to treat cancer. While chemotherapeutic drugs are designed to target rapidly dividing cancer cells, they can also affect other cell types. In the case of dermal cells and macrophages involved in wound healing, cytotoxicity often leads to the development of chronic wounds. The situation becomes even more severe when chemotherapy is combined with surgical tumor excision.

**Recent Advances:**

Despite its significant impact on patients' recovery from surgery, the issue of delayed wound healing in individuals undergoing chemotherapy remains inadequately explored.

**Critical Issues:**

This review aims to analyze the harmful impact of chemotherapy on wound healing. The analysis showed that chemotherapy drugs could inhibit cellular metabolism, cell division, and angiogenesis and lead to nerve damage. They impede the migration of cells into the wound and reduce the production of extracellular matrix. At the molecular level, they interfere with replication, transcription, translation, and cell signaling.

This work reviews skin problems that patients may experience during and after chemotherapy and demonstrates insights into the cellular and molecular mechanisms of these pathologies.

**Future Directions:**

In the future, the problem of impaired wound healing in patients treated with chemotherapy may be addressed by cell therapies like autologous keratinocyte transplantation, which has already proved effective in this case. Epigenetic intervention to mitigate the side effects of chemotherapy is also worth considering, but epigenetic consequences of chemotherapy on skin cells are largely unknown and should be investigated.

**Figure:**
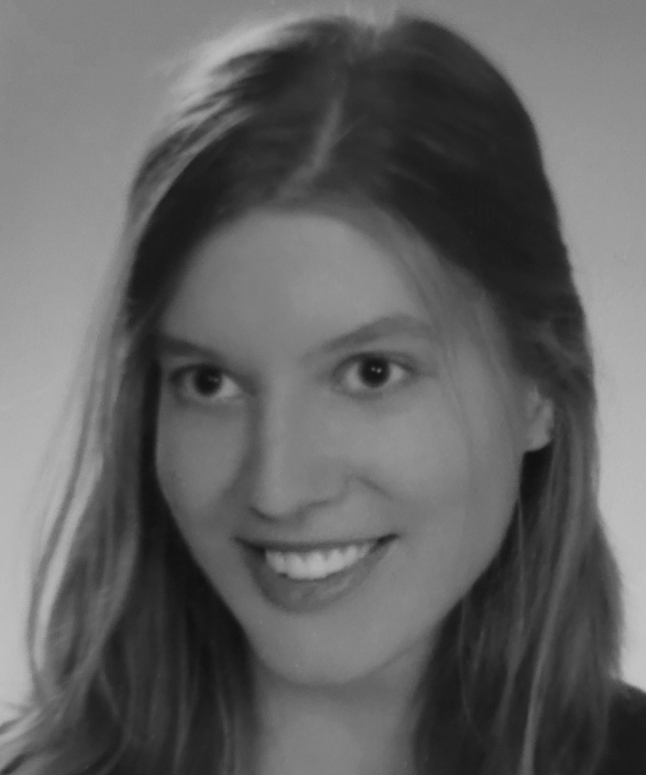
Paulina Słonimska, MSc, BE

**Figure:**
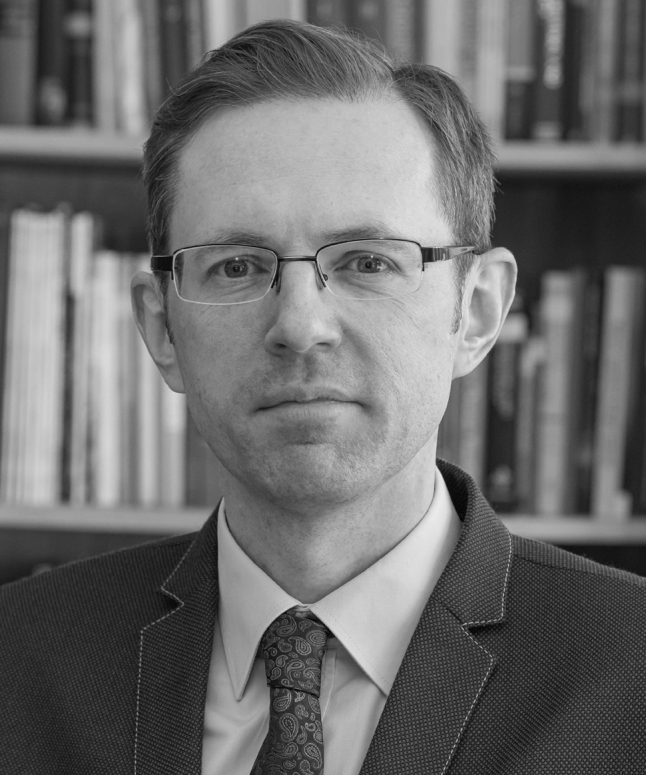
Michał Pikuła, PhD

## SCOPE AND SIGNIFICANCE

Approximately 14 million people are diagnosed with cancer each year around the world.^1^ About 30% of them are treated with chemotherapy. Chemotherapy has many severe side effects. One of them is delayed wound healing and other skin problems. This work is a collection of information about the relationship between chemotherapy and skin diseases caused by chemotherapeutic drugs on skin cells, for example, by impairing the production of growth factors.

## TRANSLATIONAL RELEVANCE

Chemotherapy can treat cancer alone or in combination with surgery and/or radiation therapy. In conjunction with surgery, it can be used preoperatively to reduce tumor mass before resection or to eliminate residual disease. Chemotherapy affecting proliferating dermal cells and macrophages impairs skin homeostasis and, thus, wound healing. In neoadjuvant chemotherapy, chemotherapeutic agents are administered before resectioning the tumor, which may lead to delayed healing of surgical wounds.^2–4^ The mechanisms involved in chemotherapy-mediated wound healing complications are poorly recognized. Research is needed to improve chemotherapy patients' quality of life and manage the frequent additional challenge presented by nonhealing wounds.

## CLINICAL RELEVANCE

There are several skin conditions associated with chemotherapy. Patients treated with chemotherapy may develop an inflammatory skin rash. Folliculitis is observed in 85% of patients receiving chemotherapy.^5^ Dryness of the epidermis may manifest even a few weeks after the treatment. Excessive dryness brings about constant pain in the fingers and cracks in the skin, increasing the risk of bacterial infections. Chemotherapy can cause hand–foot syndrome, characterized by numbness, tingling, or burning pain in the hands and feet. Some chemotherapeutics induce vein irritation, resulting in nonhealing necrotic ulcers and even nerve damage, leading to loss of limb function.

## BACKGROUND

Chemotherapeutic agents impair the pathways involved in wound healing through different mechanisms that may involve inhibiting cellular metabolism, cell division, or angiogenesis. They disrupt DNA replication, transcription, and translation. Chemotherapeutics impede cell migration into the wound, reduce extracellular matrix (ECM) production, and inhibit fibroblast proliferation.^2,3^ These deficiencies are associated with various effects, such as apoptosis, cell cycle arrest, senescence, mitotic catastrophe, inflammatory responses, and fibrosis.^4^ DNA double-strand rupture, the release of reactive oxygen species, and overall stress response are complex parts of the mechanisms leading to tissue damage.

## DISCUSSION

### Skin-affecting conditions associated with anticancer chemotherapy

**Figure 1.**
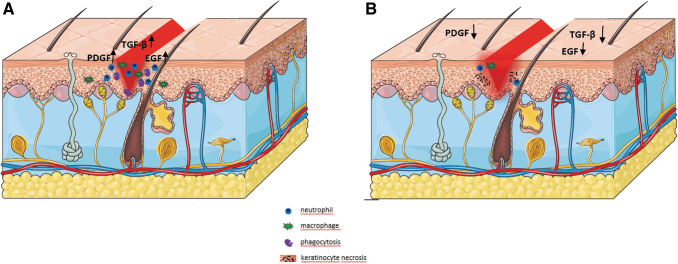
Disruption of the wound healing process under the influence of chemotherapy. **(A)** An ordinary course of the wound healing process. Inflammation in the early stages is characteristic of the high activity of neutrophils and macrophages, followed by phagocytosis of the dead tissue. Cells in the wound area synthesize PDGF, TGF-β, and EGF. **(B)** Impaired wound healing process caused by chemotherapy. Chemotherapeutic drugs suppress the immune response, thus decreasing the activity of neutrophils and macrophages, and necrosis of cells, such as keratinocytes, occurs, delaying the phagocytosis of dead tissue and thereby increasing the risk of infections.^*8*^ In addition to the reduced expression of growth factors, this leads to the formation of a chronic wound. (The drawing was created with ServierMedicalArt). EGF, epidermal growth factor; PDGF, platelet-derived growth factor; TGF-β, tumor growth factor-β.

Animal studies have shown that chemotherapy, in this case, cisplatin treatment, reduces fibroblast proliferation.^6^ As chemotherapy drugs preferentially target rapidly dividing cancer cells, they can also affect normal cells proliferating in growing tissues. Macrophages and fibroblasts involved in skin wound healing are susceptible to chemotherapy's damaging effects, similar to cancer cells.^6^ Figure 1 schematically summarizes how chemotherapy interferes with wound healing by affecting the cells involved. Several skin-affecting conditions associated with chemotherapy have been described.

MEK inhibitors are associated with an increased risk of inflammatory skin rash characterized by papulopustular eruptions and dermatitis acneiform.^7^ A rash, or folliculitis, is a typical response to systemic treatment. It occurs in 43% to 85% of patients treated with targeted systemic therapies.^5^ Xerosis cutis, a condition characterized by abnormally dry skin, may develop in 35% of patients, even several weeks after systemic therapy.^5^ Such a strong drying, in the event of an exacerbation, can cause asteatotic eczema.

It may be complicated by secondary *Staphylococcus aureus* or *Herpes simplex* infections. Xerosis cutis and the resulting eczema are often correlated with the patient's age and the tendency to develop atopic dermatitis. Very dry hand skin causes a feeling of pain in the fingers or the formation of fissures on the dorsal sides of the interphalangeal joints.^5^ Chemotherapy can also cause other discomforts to the skin and epidermis, leading to reactions such as transient erythema, discoloration, nail changes, and hand–foot syndrome. Immunosuppression is one of the side effects of chemotherapeutics. The inhibition of inflammatory response in the early stages of wound healing impairs the healing process. Reduced activity of neutrophils and macrophages increases the risk of wound infection and delays the removal of dead tissue through phagocytosis.^8^ This can often lead to a chronic wound. Chronic wounds are defined as those that do not heal for three months or more.^9^

The most severe complication is hand–foot syndrome. This condition is initially characterized by numbness in the hands and feet, tingling, or burning pain. In the advanced stage, it can cause ulceration and blisters, mainly affecting the palmar and plantar surfaces.^10,11^ It is related to the use of capecitabine and other 5-fluorouracil derivatives. The pathophysiology is not yet well characterized. However, the existing damage to the epithelial cells of the eccrine ducts suggests that basal keratinocytes may be affected.^5^

Some chemotherapy drugs induce venous irritation. These drugs are commonly known as blistering agents. They can cause severe tissue damage, leading to a nonhealing necrotizing ulcer. This kind of toxicity is demanding and requires long treatment. The characteristic symptoms include significant pain and disfigurement. In extreme cases, the condition is associated with nerve damage, which may lead to the loss of limb functionality or even amputation.^8^

A complication affecting the nails is paronychia, an inflammatory reaction of the nail folds resulting from epidermal growth factor receptor inhibitors. It is challenging to treat and leads to infections.^8^

### Effects of chemotherapy on skin cells

Chemotherapy drugs interfere with wound-healing pathways. They delay cell migration and interfere with cell proliferation.^12^ The harmful effects of chemotherapy on dermal cells are explained mainly by the sensitivity of actively proliferating cells that build the skin to cytotoxics and cytostatics and impaired production of growth factors.

Wound healing requires cellular interactions between cells such as fibroblasts, myofibroblasts, keratinocytes, smooth muscle cells, endothelial cells, and immune cells. Growth factors mediate these interactions. There is evidence that chemotherapy impairs the production of growth factors essential in skin wound healing.^13^

The growth factor families essential in wound healing include epidermal growth factors (EGF), fibroblast growth factors (FGF), insulin-like growth factor, keratinocyte growth factor (KGF), platelet-derived growth factors (PDGF), transforming growth factors (TGF), and vascular endothelial growth factor (VEGF). Varied roles of the growth factors in the wound healing processes have been identified. EGF is produced mainly by platelets and found in high concentrations in the earliest stages of wound healing. EGF increases the rate of wound epithelialization and reduces scarring, preventing excessive wound contraction. EGF can stimulate keratinocyte proliferation and migration, and KGF can then stabilize the epidermis.^14^ PDGF is stored in platelets and released in large quantities from platelet degranulation during clotting upon injury. PDGF is a potent chemoattractant of neutrophils, monocytes, and fibroblasts.

**Figure 2.**
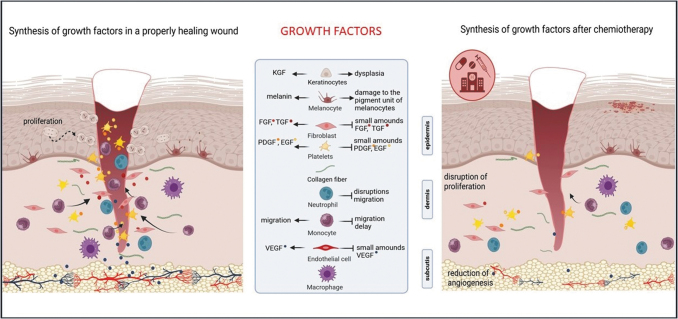
The effect of chemotherapy on skin cells by blocking the synthesis of growth factors. Chemotherapy-mediated decrease in the production of growth factors is one of the causes of complications in wound healing. In a properly progressing wound healing process, cells in and around the wound synthesize PDGF, TGF-β, and EGF. These factors, especially PDGF, are a strong attractant of neutrophils, monocytes, and fibroblasts. Chemotherapeutic agents block the synthesis of growth factors, leading to a delay in cell migration, proliferation disruption, and angiogenesis reduction. (The drawing was created with BioRender.com)

It stimulates mesenchymal cells to synthesize ECM components, collagenase, and other growth factors.^15^ VEGF is specific for endothelial cells and is a chemoattractant with an angiogenic solid effect.^14^ The role of TGF in wound healing is to promote the chemotaxis of inflammatory cells and the synthesis of ECM.^14^ Figure 2 summarizes the consequences of chemotherapy on wound healing by blocking the synthesis of growth factors. Most chemotherapy agents target actively dividing cells to destroy rapidly proliferating cancer cells. Keratinocytes are one of the most mitotically active cells in the body. Therefore, they are susceptible to the effects of most anticancer agents and their side effects.

Viable keratinocytes are sensitive to factors that are not cell cycle specific and cause the death of both dividing and quiescent keratinocytes.^16^ Severe keratinocyte dysplasia has been observed in patients after treatment with busulfan or cyclophosphamide.^17^ Chemotherapy also affects fibroblasts and melanocytes.^18^ An experiment was conducted on human cells to demonstrate the cytotoxicity of chemotherapy agents. Normal fibroblasts, melanocytes, and keratinocytes were incubated for 2, 24, and 48 h with various concentrations of cisplatin, doxorubicin, and 5-fluorouracil. All cells were most sensitive after 48 h. Incubations with doxorubicin were the most toxic.^18^

Hair follicles are the site of intensive proliferation and, thus, very susceptible to damage caused by chemotherapy. Hair loss clinically manifested as chemotherapy-induced alopecia is a common condition. Some chemotherapy drugs cause more severe hair loss, while some cause only mild or no hair loss at all. This phenomenon has not yet been investigated. Hair matrix keratinocytes are characterized by high proliferation, significantly below the line of mature hair follicles, and chemotherapy causes extensive cell death in this region. In addition to activating apoptosis, chemotherapy disrupts the terminal differentiation program in hair follicle keratinocytes. Chemotherapy also harms melanocytes of the pigment unit of hair follicles.^4^

### Effect of chemotherapy on dermal stem cells

Stem cells are responsible for tissue renewal in the human body throughout life. Each organ, including the skin, contains a specific population of stromal cells varying in their differentiation potential. Skin stem cells maintain homeostasis and regulate skin damage under physiological conditions.^19^ Research data on the effects of chemotherapy drugs on the stem cell types found in the skin are available for mesenchymal stromal stem cells (MSCs), epidermal stem cells (EPSCs), and hair follicle stem cells (HFSCs).

MSCs contribute to structural tissue repair and have potent immunomodulatory and anti-inflammatory properties. Modulating the local environment may affect tissue repair.^20^ MSCs form a heterogeneous population of multipotent stromal cells and have regenerative capabilities attributed to their ability to differentiate into functional cells. MSCs are sensitive to the effects of chemotherapy, dependent on the class of chemotherapeutics. MSCs are resistant to platinum-based compounds and cyclophospase inhibitors. Melphalan, an alkylating agent, reduces MSC's proliferation but does not affect their viability, while another alkylating agent, busulfan, induces MSC's apoptosis. MSCs can avoid chemotherapy-induced apoptosis, which is attributed to paracrine signaling in the stem cell microenvironment rather than to the repair of individual cell DNA damage.^21,22^

Experiments in the model of MSCs obtained from bone marrow from adults may shed light on mechanisms underpinning the action of chemotherapeutics on stem cells. The cells were treated with doxorubicin and etoposide at 10 nM and 500 ng/mL, respectively, the concentrations causing the breaking of the double-stranded DNA. The treatment resulted in significant shortening of telomeres after 5 days of exposure to the drug combination; the stem cells could not recover the lost telomere sequences for up to 28 days of culture, similar to naturally aging stem cells.

Chemotherapy-induced telomere shortening was associated with disrupting the proliferative and differentiation potential and accelerated adipose tissue differentiation.^23^ Doxorubicin, etoposide, and camptothecin are the chemotherapeutic agents that induce DNA double-strand breaks, in this manner interfering with topoisomerases' ability to religate DNA, and thus leading to cellular senescence.^24–26^

EPSCs are an extensive group of skin stem cells. There are no studies yet showing the effects of chemotherapy on EPSCs. However, because EPSCs can proliferate,^27^ they are exposed to chemotherapy-like keratinocytes and other actively dividing cells.

HFSCs have been reported to accelerate cutaneous wound healing.^28^ HFSCs in the hair bulge have the potential to differentiate into epidermal lineages such as keratinocytes.^29^ In contrast to the immediate, destructive changes in rapidly proliferating hair matrix cells, quiescent HFSCs show massive proliferation after busulfan chemotherapy. However, under the influence of cyclophosphamide, they undergo apoptosis. The findings of Kim et al. showed that stem cells lose their resistance to DNA damage, resulting in permanent loss of regeneration after alkylating chemotherapy.^30^

### Examples of chemotherapy agents and their effects on wound healing

Different effects on cancer cells and wound healing characterize several classes of chemotherapeutic agents. Alkylating agents such as cyclophosphamide (Cytoxan), chlorambucil (Leukeran), thiotepa (Tioplex), mechlorethamine (Mustargen), and cisplatin (Platinol) inhibit the cell cycle by alkylating DNA nucleotides, leading to crosslinking, strand breakage, and RNA miscoding. The agents mentioned above have varied effects on wound healing.^6^ Cyclophosphamide inhibits wound healing by reducing the initial vasodilatation and subsequent neovascularization during the proliferative phase of wound healing.^31^ Studies in animal models show reduced wound tensile strength at doses from 165 to 500 mg/kg.^6,31^

Mechlorethamine harms the functions of fibroblasts, which delays wound healing. Administration of this agent at the time of injury leads to histological signs of impaired healing, including delayed fibroplasia, endothelial proliferation, and the production of extracellular fibers. In animal studies, doses of 0.3 to 0.6 mg/kg reduced the tensile strength of the wound.^6^

Cisplatin was found to inhibit the early proliferative phase of wound healing.^32^ This finding aligns with the studies demonstrating that with a single dose of 5 mg/kg cisplatin to rats, the wound reduced tensile strength on days 4, 7, 14, and 28 after surgery.^32^

Methotrexate is an agent often used in the treatment of tumors.^33^ Methotrexate reduces cell proliferation by inhibiting dihydrofolate reductase, essential for purine synthesis. It is then administered in very high doses. Five percent of cancer patients receiving high doses of methotrexate experience skin erosions.^33^ Samples from the skin biopsy of patients treated with a high dose of methotrexate showed numerous keratinocyte dystrophies, such as impaired keratinocyte maturation, widening intercellular spaces, irregular large nuclei, and apoptosis.^33^ Complications in wound healing have been observed in patients treated with chemotherapy based mainly on adriamycin with the addition of mesna, ifosfamide, and dacarbazine. They occurred in 14 of 48 patients (29%). Of these 14 patients, 12 received antibiotics for wound infection, 10 required hospitalizations, and 9 underwent reoperation.^34^

The commonly used chemotherapy with actinomycin D has been shown to be detrimental to the skin in animal models. Postoperative administration of actinomycin intraperitoneally at a dose of 0.6 mg/kg to mice resulted in a 38% decrease in the tensile strength of the wound on the third postoperative day.^6^

Examples of chemotherapy agents exerting negative effects on the skin are presented in Table 1.

**Table 1.** Examples of chemotherapy agents used in cancer treatment known for side effects on skin and wound healing

Chemotherapy Drug	Mechanism of Action	Indication	Complication
Cyclophosphamide^6^	Alkylation of DNA^31^	Multiple myeloma, sarcoma, and breast cancer^31^	Reduction of initial vasodilation and subsequent neovascularization during the proliferative phase of wound healing, thereby inhibiting the wound-healing process^6,27^
Mechlorethamine^6^	Alkylation of DNA^32^	Cutaneous T cell lymphoma^32^	Delayed fibroplasia, endothelial proliferation, and the production of extracellular fibers^6^
Cisplatin^28^	Crosslinking with purine bases in DNA, interfering with DNA repair mechanisms, causing DNA damage, and then inducing apoptosis in cancer cells^33^	Bladder, head and neck, lung, ovarian, and testicular cancers^33^	Inhibiting the early proliferative phase of wound healing^28^
Methotrexate^29^	Reduction of cell proliferation by inhibiting dihydrofolate reductase, essential for purine synthesis^29^	Breast cancer^34^	Skin erosions; keratinocyte dystrophies^29^
Adriamycin^30^	The induction by adriamycin of strand breaks in the DNA of L1210 leukemic cells^35^	Leukemia^35^	Impaired wound healing^30^
Actinomycin D^6^	Ability to intercalate into the DNA duplex with high affinity, thereby interfering with DNA replication and transcription^36^	Cervical carcinoma and breast cancer^36^	Impaired wound healing^6^
Oxaliplatin^37^	G2/M arrest and apoptosis are characterized by the translocation of Bax into the mitochondria and the release of cytochrome c into the cytosol^38^	Resected stage II colon cancer^38^	Sensory neuropathy; hypersensitivity^39^
Entrectinib^75^	Receptor inhibition of tropomyosin tyrosine kinases (TRKs) TRKA, TRKB, TRKC, as well as proto-oncogenic protein tyrosine kinase ROS1 and anaplastic lymphoma kinase (ALK)^76^	Pancreatic cancer^40^	Neuropathic arthropathy: foot pain, swelling, and sensory changes; dermatological toxicity in the form of a rash, skin pain, and itching^76^
Olaparib^41^	Inhibition poly(ADP-ribose) polymerase, thereby blocking the repair of single-strand DNA breaks. This results in synthetic lethality in BRCA1-associated cancer cells, which have dysfunction of another DNA repair pathway homologous recombination^41^	Pancreatic cancer^42^	Dorsal dermatosis of the hand^43^
Larotrectinib^44^	Binding to Trk, thereby preventing neurotrophin–Trk interaction and Trk activation, which results in both the induction of cellular apoptosis and the inhibition of cell growth in tumors that overexpress Trk^44^	Pancreatic cancer^45^	Impairment of wound healing^45^
Capecitabine^46^	inhibition of the cytochrome P-450 isoenzyme system 2C9^47^	Breast cancer; resected biliary tract cancer^48^	Hand–foot syndrome^49^

### The effect of chemotherapy on blood and lymphatic vessels and angiogenesis

Angiogenesis, or the growth of blood vessels, is essential for tissue growth. An imbalance in this process contributes to numerous malignant, inflammatory, ischemic, infectious, and immune disorders.^35^ Dysregulation of angiogenesis is the driving force behind many serious diseases, including cancer. Tumor angiogenesis is essential for delivering oxygen and nutrients to the growing tumor. Angiogenesis alone does not initiate malignancy but promotes tumor progression and metastasis.^35,36^

Angiogenesis also plays an essential role in wound healing. During this process, angiogenic capillary sprouts penetrate the fibrin or fibronectin-rich wound clot and organize it into a microvascular network throughout the granular tissue.^37^ As collagen accumulates in the granulation tissue to form scar tissue, the density of blood vessels decreases. There is a dynamic interaction between endothelial cells, angiogenic cytokines (such as FGF, VEGF, TGF-β), angiopoietin, and mast cell tryptase, and the ECM environment. Specific endothelial cell ECM receptors are critical to these morphogenetic changes in blood vessels during wound healing.

**Figure 3.**
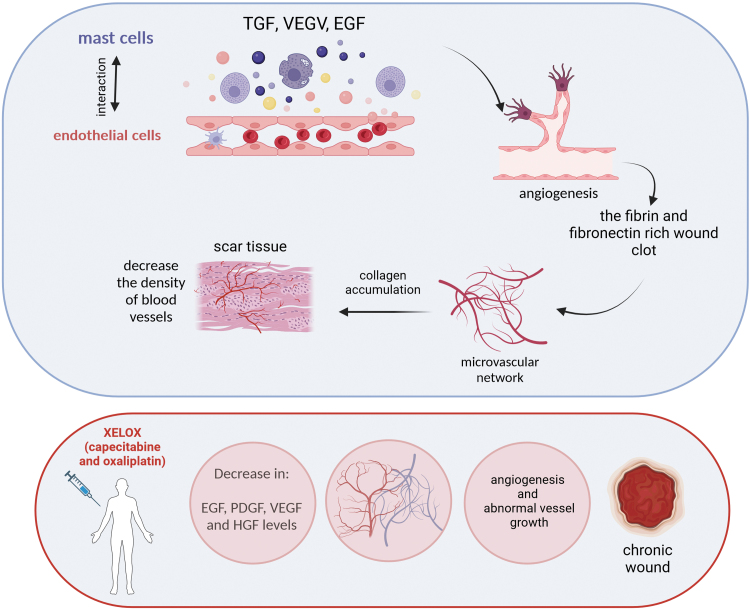
The role of angiogenesis in wound healing and its disruption by agents used in chemotherapy. The effect of angiogenesis on wound healing. Angiogenic capillary sprouts penetrate the clot rich in fibrin and fibronectin, organizing themselves into a microvascular network. Collagen accumulating in the granulation tissue contributes to scar tissue formation; the density of blood vessels is reduced. Angiogenesis is significantly influenced by the interaction between endothelial cells, angiogenic cytokines (FGF, VEGF, TGF-β), angiopoietin, mast cell tryptase, and the ECM environment. Patients after treatment with XELOX, that is, a mixture of capecitabine and oxaliplatin, showed a decrease in the levels of EGF, PDGF, VEGF, and HGF, which may block the proper course of angiogenesis and lead to the formation of a chronic wound. (The drawing was created with BioRender.com). ECM, extracellular matrix.

Wound angiogenesis is likely regulated by the interaction of endothelial cells with the specific three-dimensional environment of the ECM in the wound space.^37^ These interactions are schematically shown in Fig. 3. The reduced vascular growth is a crucial aspect of many nonhealing wounds.^38^ Er et al. measured the plasma levels of angiogenic factors in patients with metastatic colorectal cancer before and after chemotherapy in three cycles of XELOX, that is, capecitabine and oxaliplatin.^39^

Thirty-eight patients were included in the study,^39^ showing a statistically significant decrease in EGF, PDGF, VEGF, and HGF levels^39^ (Fig. 3). Conventional cytotoxic anticancer drugs have antiangiogenic effects,^40^ thereby blocking tumor growth. Therefore, antiangiogenic properties of chemotherapeutics may be one of the primary causes of hindered wound healing after chemotherapy. However, no studies currently address chemotherapy's effects on wound healing by reducing angiogenesis or vessel damage.

### Chemotherapy-induced peripheral neuropathy and impaired skin wound healing

Peripheral neuropathies are often consequences of chemotherapy. A meta-analysis of 31 studies estimated that the incidence of peripheral neuropathies is 68.1% in the first and 30.0% in the sixth month following chemotherapy.^41^ Peripheral neuropathies are associated with chronic pain that dangerously reduces the patient's overall condition. Impaired healing of surgical wounds is another complication observed in oncological patients following radio and chemotherapy.^6^ The connection between neuropathies and impaired wound healing is well recognized in diabetes.^42^ Neuropathy is diagnosed in around 50% of patients with diabetic foot syndrome.^43^ High doses, several treatment courses, combination chemotherapy, age, diabetes, vitamin deficiencies, or preexisting peripheral neuropathies increase the risks of chemotherapy-induced peripheral neuropathy (CIPN).^44^

Whether CIPN contributes to delayed wound healing in oncological patients needs to be addressed in research. However, denervation experiments in animal models demonstrate that skin wound healing is nerve dependent.^45,46^ The inhibitory effect of skin denervation on wound healing is associated with the loss of secretion of neuropeptides, such as substance P.^47^

It is worth noting that sensory nerves were implicated in promoting reepithelialization through substance P secretion,^48^ a critical step of wound closure.

Oncological drugs associated with peripheral neuropathies include platinum derivatives (*e.g.,* oxaliplatin), taxanes (*e.g.,* paclitaxel), vinca alkaloids (*e.g.,* vincristine), immunomodulators (thalidomide), proteasome inhibitors (bortezomib), monoclonal antibodies (immune checkpoint inhibitors like, *e.g.,* ipilimumab).^49^ The neurotoxic actions affect myelin sheets (myelinopathy), sensory cell bodies in the dorsal root ganglion (neuronopathy), axon components (axonopathy), and nerve endings.^50^ The mechanisms of chemotherapy-induced neurotoxic effects are diverse and depend on the drugs' mechanism of action.

The anticancer drugs causing CIPN are known to target microtubules (paclitaxel and vincristine), mitochondria (oxaliplatin, bortezomib, paclitaxel), and DNA (oxaliplatin, cisplatin). A number of alterations at the molecular and cellular levels are observed in CIPN, including changes in voltage-gated ion channels and neurotransmitters such as elevated glutamine levels, increased ROS release due to mitochondrial dysfunction, induction of MAPK pathway, and elevated cytokines (IL8, IL1B, TNFA, MCP1).^51^

The neurotoxic effects include defective axonal transport^52^ and altered inositol-triphosphate receptor phosphorylation, resulting in intracellular calcium flux.^53^

Other chemotherapy consequences causing nerve injuries are capillary damage,^50^ neuroinflammation,^54^ swelling, and fibrosis, leading to compression of peripheral nerves.^55^ Axonal damage in CIPN occurs through Wallerian degeneration but also apoptosis. Microtubule stabilization impairing its functions is implicated in inducing Wallerian degeneration, while apoptosis is connected with the destruction of mitochondria, DNA damage, neuroinflammation, and impaired cellular signaling.^56^ Demyelination and decreased density of intraepidermal nerve fibers are typical consequences of CIPN.^57^ Demyelination involves the dissociation of Schwann cells from nerve fibers. Experimental data suggest that Schwann cells' dedifferentiation following low-dose paclitaxel treatment is one of the demyelination mechanisms.^58^

**Figure 4.**
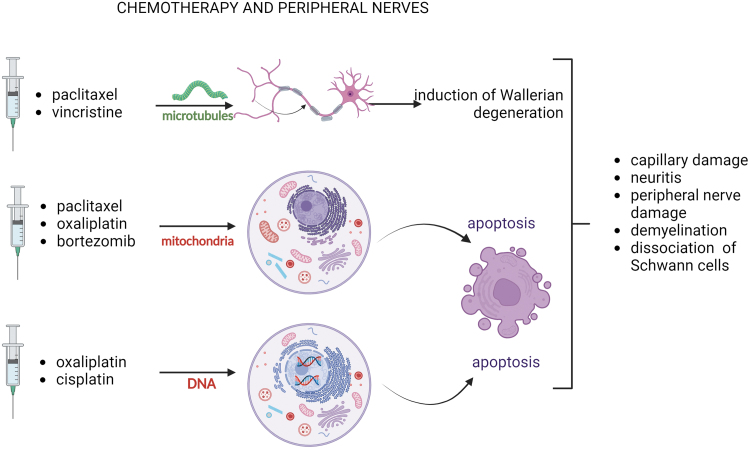
Effects of chemotherapeutic on peripheral nerves. CIPN inducing anticancer drugs attack microtubules, mitochondria, and DNA. Axonal damage in CIPN occurs through Wallerian degeneration, associated with microtubule stabilization and apoptosis, which involves mitochondrial destruction and DNA damage. These injuries lead to complications in the nervous system, such as capillary damage, neuritis, peripheral nerve damage, and demyelination, including dissociation of Schwann cells. (The drawing was created with BioRender.com). CIPN, chemotherapy-induced peripheral neuropathy.

Effective pharmacological solutions for peripheral neuropathies are lacking. The list of drugs indicated to treat the effects of peripheral neuropathies includes SSNRI (serotonin and norepinephrine reuptake inhibitors, *e.g.,* Duloxetine), antidepressants (*e.g.,* Amitriptyline), anticonvulsants (*e.g.,* Gabapentin, Pregabalin), and opioids. Based on existing data, a weak recommendation for Duloxetine can be suggested.^50^ The use of vitamin B complex and nutraceuticals, alpha-lipoic acid, L-acetylcarnitine, vitamin E, and Coenzyme Q is reported in treating polyneuropathies.^59^ The neurotoxic effects of selected chemotherapeutics are summarized in Fig. 4. Recognizing that skin wound healing is nerve dependent, it is worth considering that chemotherapy-induced nerve injuries could be one of the primary causes of impaired wound healing, yet it needs direct evidence.

### Effects of chemotherapy on the immune system in the context of cutaneous wound healing

Chemotherapy profoundly affects the immune system,^60^ which in turn plays a crucial role in responding to injury. In particular, chemotherapy-induced immunosuppression can seriously impair skin wound healing. Chemotherapy-induced immunosuppression involves diverse mechanisms. Those include lymphodepletion and reduced numbers of immunosuppressive cells, inflammation, and activation of effector cells. Chemotherapy impairs the function and lowers the number of T cells, natural killer cells, and dendritic cells.

Chemotherapy can also alter the differentiation and maturation of immune cells, resulting in dysfunctional immune responses.

Moreover, chemotherapy can disrupt communication and signaling pathways between immune cells.^47,61^ A severe consequence of decreased neutrophil and macrophage activity is delayed removal of dead tissue and foreign bodies from the wound, which increases the risk of infections and thus complicates healing.^8^ In such conditions, infections can spread rapidly, leading to sepsis.^62^

Recent studies report that the clinical efficacy of chemotherapy may also be attributed to the restoration of immune surveillance. Significantly, combination chemotherapy increases tumor recognition and elimination by the host immune system while reducing immunosuppression from the tumor microenvironment. For example, cisplatin or oxaliplatin may demonstrate anticancer effectiveness by inducing type I IFN and IFN signaling and enhancing tumor recognition by T cells.^63^

It is also worth mentioning that chemotherapy has been connected with chemotherapy-induced autoantibodies^64^ and other chemotherapy-induced autoimmune reactions, including those affecting the skin, like lupus.^65^ However, such reports are rare.

### Epigenetic consequences of chemotherapy and epigenetic intervention as a method supporting chemotherapy

Epigenetic mechanisms initiate and maintain hereditary patterns of gene function and regulation without affecting the nucleotide sequence of the genome. Epigenetic mechanisms include covalent modifications of histones, such as methylation and acetylation; modifications of DNA, such as methylation; and chromatin remodeling. Abnormal epigenetic regulation significantly impacts oncogenes' expression and ultimately may lead to cancer. Recent studies report that epigenetic deregulation may not only be involved in cancerogenesis, but it can affect pharmacological treatment by modulating the expression of genes involved in the absorption, distribution, metabolism, and excretion of drugs, thus contributing to variability in drug response and side effects. For example, specific DNA methylation patterns can be used as a biomarker to predict response to chemotherapy.^66^

While epigenetic therapies are being researched to reverse epigenetic changes leading to chemotherapy resistance,^67^ studies to improve skin wound healing using epigenetic drugs are rare but worth the attention; for example, zebularine, a DNA methyltransferase inhibitor, was shown to promote ear pinna hole closure in mice, which involved skin restoration.^68^ Although the evidence for mitigating the side effects of epidrug-mediated chemotherapy is scarce, the experiments described above seem to be a direction deserving further research.

Recently, a study has been carried out showing that epigenetic therapy can support neoadjuvant chemotherapy in breast cancer and improve the healing process of postoperative wounds.^69^ Triple-negative breast cancer (TNBC) cells were treated with paclitaxel alone or in combinations with epigenetic drugs: suberoylanilide hydroxamic acid, an inhibitor of histone deacetylases, and azacytidine, an inhibitor of DNA methyltransferases. The combination of epigenetic drugs increased toxicity to TNBC cells compared with paclitaxel alone. As demonstrated by a scratch assay, adipocyte stem cells preconditioned with a combination of paclitaxel with the epigenetic drugs showed an improved healing effect on cultured fibroblasts compared with those preconditioned with paclitaxel alone.^69^

## SUMMARY

Chemotherapeutics exert cytotoxic and cytostatic actions on cancer cells but also inhibit wound healing. Chemotherapeutic agents inhibit cellular metabolism, cell division, and angiogenesis and damage nerves. They disrupt the processes of replication, transcription, and translation. They also hinder cell migration to the wound and reduce ECM production. There is also inhibition of fibroblast proliferation and keratinocytes.

**Figure 5.**
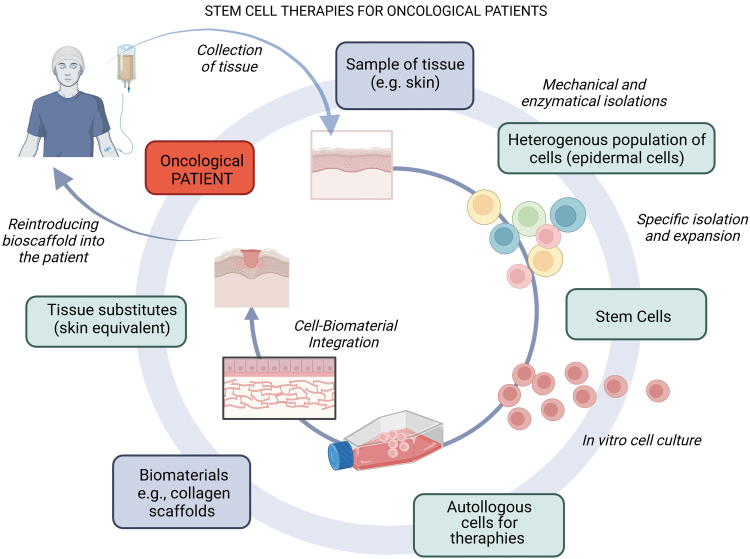
Cell therapy strategy for oncology patients. Tissue obtained from an oncological patient, such as skin, is transported to a laboratory, where stem cells are isolated for cell culturing. Through appropriate *in vitro* propagation, the cells can be combined with biomaterial to create bioscaffolds. These ATMPs can be applied to hard-to-heal wounds or skin losses. This strategy capitalizes on the innate regenerative capacity of the patient's cells, amplified and guided by the tissue-engineered bioscaffold. (The drawing was created with BioRender.com). ATMP, advanced therapy medicinal product.

Chemotherapy-mediated complications of skin wound healing are not well recognized, and based on our literature research, no treatments dedicated to preventing and mitigating such side effects are available. In severe cases, cell-based therapies have been reported.^70–72^ An exemplary cell therapy strategy for oncology patients is schematically shown in Fig. 5.

Although clinically approved cell-based therapies are indicated for treating burns and diabetic foot (Apligraf, Dermagraft), only preliminary trials to support skin wound healing complications following chemotherapy were recorded^70,71^ Autologous keratinocyte transplantation appears to be a challenging but promising solution to treat delayed wound healing caused by chemotherapy. Langa et al. demonstrated that autologous cells from a cancer patient suspended in fibrin sealant and transplanted directly into the nonhealing wound, closed the wound within two months.^72^ The transplanted keratinocytes stimulated wound healing by forming the epidermis and secreting growth factors and cytokines. The use of autologous cells essentially decreases the risk of graft rejection. However, the approach requires *in vitro* cell expansion, and the *in vitro* conditions may affect the transplantation potential.^73^

Other than treating the side effects of chemotherapy of hindered healing, the issue is stimulating the healing of difficult-to-heal wounds. Adipose-derived mesenchymal stromal cells (AD-MSCs) can be used for this purpose. They are easy to obtain from adipose tissue, even in large amounts, and show great pro-regenerative potential through the paracrine effect^.^^74^

Epigenetic mechanisms are known to regulate the expression of genes responsible for tumor formation and influence drug treatment by modulating genes involved in drug distribution and absorption. The epigenetic consequences of chemotherapy on dermal cells and wound healing are poorly recognized, but epigenetic intervention is worth considering to mitigate the side effects of chemotherapeutics.

Although wound healing complications as side effects of chemotherapy are not critical in cancer treatment, they are worth considering and exploring more profoundly. Although understudied, the formation of chronic wounds after chemotherapy treatment is a significant issue. It affects patients with combined therapy––tumor removal and chemotherapy, for example, breast cancer, who often have to deal with both chemotherapy and surgery side effects. In such cases, healing wounds after mastectomy may become complicated. Impaired wound healing may also manifest itself long after oncological treatment, making it impossible or difficult to perform other surgical procedures.

**TAKE HOME MESSAGES**

- Chemotherapy is a primary method to treat cancer, but chemotherapeutics can impair skin homeostasis
- Chemotherapeutic drugs can affect actively proliferating cells, including dermal cells and macrophages involved in wound healing
- Disruption of skin homeostasis is particularly problematic for surgical patients, as it often leads to the development of chronic wounds
- Chemotherapy can lead to various skin problems, such as rash, folliculitis, hand–foot syndrome, venous irritation, paronychia
- Chemotherapy-mediated complications of skin wound healing are not well recognized, and no treatments dedicated to preventing and mitigating such side effects are available

## ABOUT THE AUTHORS

**Paulina Słonimska, MSc, BE:** conceptualization, review and editing, and writing––original draft preparation; **Paweł Sachadyn, PhD:** conceptualization, writing––original draft preparation, supervision, funding acquisition; **Jacek Zieliński, PhD, MD:** conceptualization, review, and editing; **Marcin Skrzypski, PhD, MD:** review and editing; **Michał Pikuła, PhD:** conceptualization, review and editing, funding acquisition, and supervision.

## Abbreviations and Acronyms

AD-MSCs: adipose-derived mesenchymal stromal cells
ALK: anaplastic lymphoma kinase
CIPN: chemotherapy-induced peripheral neuropathy
ECM: extracellular matrix
EGF: epidermal growth factor
EGFR: epidermal growth factor receptor
EPSC: epidermal stem cells
FGF: fibroblast growth factor
HFSC: hair follicle stem cells
IFSC: interfollicular stem cells
IFN: interferon
IFN-κ: interferons κ
IRF1: factor 1 interferon
KGF: keratinocyte growth factor
MAPK: mitogen-activated protein kinase
MEK: mitogen-activated protein
PDGF: platelet-derived growth factor
ROS1: proto-oncogene tyrosine-protein kinase
SSNRI: serotonin and norepinephrine reuptake inhibitors
TGF-β: tumor growth factor-β
TNBC: triple-negative breast cancer
TRK: tropomyosin tyrosine kinase
VEGF: vascular endothelial growth factor
